# Supplementary material for: Low-Frequency Electrical Stimulation of the Auricular Branch of the Vagus Nerve in Patients with ST-Elevation Myocardial Infarction: A Randomized Clinical Trial
Source: J Clin Med. 2025 Mar 10;14(6):1866. doi: 10.3390/jcm14061866 (PMC11943318; doi:10.3390/jcm14061866)
Supplement: Supplementary file 1 [file jcm-14-01866-s001.zip › jcm-3496455-supplementary.pdf]

NCT05992259

Auricular Vagus Stimulation and STEMI

S1. Drug therapy in hospital

Parameter	Active tVNS (n = 54)	Sham tVNS (n= 55)	p
Aspirin, %	100	100	1.00
Ticagrelorol, %	89	85	0.745
Clopidogrel, %	9	10	0.812
Glycoprotein IIb/IIIa inhibitors, %	6	11	0.199
NOAC, %	4	10	0.153
NSAIDS, %	98	81	0.010*
ACE inhibitors/Sartans, %	100	93	0.545
Amiodarone, %	7	24	0.016*

Note: NOACs, novel oral anticoagulants; NSAIDs, non-steroidal anti-inflammatory drug; ACE, Angiotensin-converting-enzyme. * - statistically significant differences.
